# Phosphatidylinositol 3'-kinase, mTOR, and Glycogen synthase kinase-3β mediated regulation of p21 in human urothelial carcinoma cells

**DOI:** 10.1186/1471-2490-11-19

**Published:** 2011-08-24

**Authors:** Nicole L Yohn, Caitlyn N Bingaman, Ashley L DuMont, Lina I Yoo

**Affiliations:** 1 Department of Biology, Denison University, 100 W. College St., Granville, OH 43023, USA

## Abstract

**Background:**

The PTEN/Phosphatidylinositol 3'-kinase (PI3-kinase) growth factor signaling pathway plays a critical role in epithelial tumor development in a multitude of tissue types. Deletion of the *Pten *tumor suppressor gene in murine urothelial cells *in vivo *results in upregulation of cyclin-dependent kinase inhibitor p21. We have previously shown in mice that p21 expression blocks an increase in urothelial cell proliferation due to *Pten *deletion. In this study, we utilized human urothelial carcinoma cells UMUC-3 and UMUC-14 to identify the signaling pathways downstream of PI3-kinase that regulate p21.

**Methods:**

Cells were treated with a combination of PI3-kinase stimulating growth factors and kinase inhibitors, or transfected with exogenous genes in order to identify the signaling events that are necessary for p21 induction. Mice with conditional deletion of *Pten *in bladder urothelium were also examined for evidence of PI3-kinase pathway signaling events that affect p21 expression.

**Results:**

When cells were treated with PI3-kinase activating growth factors EGF or PDGF, we found that p21 levels increased, in a manner similar to that observed in mice. We used the inhibitors LY294002, Akti-1/2, and rapamycin, to show that p21 induction is dependent upon PI3-kinase and AKT activity, and partially dependent on mTOR. We treated the cells with proteasome inhibitor MG-132 and found that p21 may be degraded in the proteasome to regulate protein levels. Importantly, our findings show that GSK-3β plays a role in diminishing p21 levels in cells. Treatment of cells with the GSK-3β inhibitor SB-216763 increased p21 levels, while exogenous expression of GSK-3β caused a decrease in p21, indicating that GSK-3β actively reduces p21 levels. We found that a combined treatment of LY294002 and SB-216763 improved the cytotoxic effect against UMUC-3 and UMUC-14 carcinoma cells over LY294002 alone, suggesting potential therapeutic uses for GSK-3β inhibitors. Immunohistochemical staining in bladders from wild-type and *Pten*-deleted mice indicated that GSK-3β inhibitory phosphorylation increases when *Pten *is deleted.

**Conclusion:**

PI3-kinase and AKT cause an upregulation of p21 by suppressing GSK-3β activity and activating mTOR in both cultured human urothelial carcinoma cells and mouse urothelial cells *in vivo*.

## Background

It has been well established that the phosphatase and tensin homologue deleted on chromosome 10 (*PTEN) *gene plays an important role in suppressing tumor development in multiple human cell types and organs such as the endometrium, brain, skin, and prostate [1]. Studies in the last few years have shown that *PTEN *mutation is also associated with bladder cancer [2,3]. Multiple studies utilizing tissue microarray analysis and immunohistochemistry have shown that PTEN expression is diminished in late bladder cancers of higher tumor stage and grade [4-7]. Screens of human bladder cancer cell lines have also revealed that PTEN expression is often lost [8-10]. Exogenous expression of PTEN in bladder cancer cells results in decreased invasiveness [11], providing an explanation for why PTEN loss in advanced cancers is common. The finding that PTEN expression is reduced in bladder cancer is consistent with PTEN's known functions not only in inhibiting cell migration but also in suppressing cell proliferation and apoptosis, as well as maintaining genomic integrity [1].

The principal manner in which PTEN appears to suppress cell growth is through its lipid phosphatase activity [12]. PTEN removes the phosphate from the D3 position of phosphatidylinositol-3,4,5-trisphosphate (PIP_3_) to generate phosphatidylinositol-4,5-bisphosphate (PIP_2_) [13]. The reverse reaction is catalyzed by Class I phosphatidylinositol 3-kinases (PI3-kinases) in response to activation by receptor tyrosine kinases and G-protein coupled receptors [14]. PIP_3 _in the plasma membrane generated by PI3-kinase leads to the recruitment and activation of the AKT serine/threonine kinase [15]. AKT in turn phosphorylates numerous substrates that lead to cell proliferation, growth, and survival. One known substrate of AKT is glycogen synthase kinase-3 beta (GSK-3β) [16], a serine-threonine kinase that plays an important role in insulin signaling. Phosphorylation of GSK-3β by AKT inactivates its kinase activity [16]. There is a second isoform of glycogen synthase kinase called GSK-3α that is also inhibited by phosphorylation by AKT, but its function is less clear [17]. Another important kinase that is activated downstream of AKT is mTOR; it mediates an increase in protein synthesis and cell growth, among other functions [18].

In a previous study, we generated mice in which *Pten *was conditionally deleted in bladder urothelium in order to study the effects on tumorigenesis and PI3-kinase signaling [19]. Notably, we found that the cyclin-dependent kinase p21 was consistently upregulated in the PTEN-deficient cells. As in normal urothelial cells, the p21 remained in the nucleus, indicating that activation of the PI3-kinase pathway was not leading to relocalization of p21 to the cytoplasm [20]. Other subsequent studies in the liver [21] and in kidney cells [22] have also shown that activation of the PI3-kinase/AKT pathway and PTEN knockdown lead to an increase in p21 levels.

This increase in p21 levels is important because it suppresses bladder urothelial proliferation elicited by the *Pten *deletion [19], and may contribute to reduced tumorigenesis in the bladder. The p21 protein inhibits cell proliferation by functioning as a cyclin-dependent kinase inhibitor [23], and p21 exhibits tumor suppressor functions as shown by the finding that p21-/- 129Sv/C57Bl6 mice develop spontaneous tumors at 16 months of age [24]. Immunohistochemical studies of human tumors suggest that the p21 induction we observed in mice may occur in human bladder cells as well, since p21 levels are increased in bladder tumors [25,26] and in transitional cell carcinoma cell lines [27] compared to normal urothelium. Importantly, bladder tumors with increased grade [25,28] and/or stage [25,29] have reduced p21 levels compared to lower grade or stage noninvasive tumors, suggesting that p21 expression is selectively lost in advanced tumors. Furthermore, tumors that have lost p21 expression are associated with decreased probability of survival [30].

In many cell types, PI3-kinase/AKT signaling leads to increased cell proliferation, so the fact that it induces p21 and inhibits cell proliferation in bladder urothelial cells is surprising. The commonly accepted model that PI3-kinase/AKT signaling induces cell cycle progression does not apply to urothelial cells. Understanding how urothelial cells will respond to stimulation or inhibition of this signaling pathway is important for tailoring therapy for tumors originating from the urothelium. We therefore aimed to elucidate the mechanism by which PI3-kinase/AKT signaling leads to p21 increase in human urothelial cells.

## Methods

### Cell culture

Human UMUC-3 urinary bladder transitional cell carcinoma cells were obtained from the American Type Culture Collection (Manassas, VA). The cells were passaged in DMEM (Hyclone) supplemented with 10% newborn calf serum (Hyclone), penicillin (100 U/ml) and streptomycin (100 mg/L) (Sigma) in a humidified incubator containing 5% CO_2 _and maintained at 37°C. Human UMUC-14 urothelial carcinoma cells were generously provided by Herbert Grossman (MD Anderson Cancer Center). These cells were maintained in DMEM with 10% fetal calf serum (Hyclone) and penicillin (100 U/mL)/streptomycin (100 mg/L).

### Materials

PDGF-BB and EGF were obtained from Peprotech (Rocky Hill, NJ). LY294002, MG-132, and SB-216763 were purchased from Enzo Life Sciences (Plymouth Meeting, PA). Akti-1/2 was purchased from EMD Biosciences (San Diego, CA). Rapamycin was obtained from LKT Labs (St. Paul, MN). The monoclonal p21 antibody was obtained from BD Biosciences (San Diego, CA). The phospho-AKT ser 473, GAPDH, β-catenin, GSK-3α, GSK-3β, and phospho-GSK-3 α/β ser 9/21 antibodies were purchased from Cell Signaling Technology (Danvers, MA). The 12G10 α- tubulin and α-actin antibodies were obtained from the Developmental Studies Hybridoma Bank (University of Iowa). The anti-phospho-Histone H3 antibody was obtained from Upstate (Lake Placid, NY). The mammalian expression vectors GSK3 alpha pMT2 (15896) and HA GSK3 beta wt pcDNA3 (14753) were purchased from Addgene (Cambridge, MA). siRNA (siGENOME smartpool) was purchased from Dharmacon (Lafayette, CO).

### Western blotting

Cells were treated as described and then washed in phosphate-buffered saline. Cells were lysed in ice-cold RIPA buffer (50 mM Tris pH 7.5, 1% NP-40, 150 mM NaCl, 1 mM EDTA pH8, 1 mM PMSF, 1 mM sodium orthovanadate, 1 mM sodium fluoride). After centrifugation at 13,000 rpm for 5 min, supernatants were isolated, and an aliquot was measured for protein concentration using a BCA Assay (Pierce). The lysates were mixed in a 1:1 ratio with 2 × electrophoresis sample buffer (125 mM Tris-Cl pH 6.8, 20% glycerol, 4% SDS, 10% beta mercaptoethanol, 0.0025% bromophenol blue). Approximately 25 μg of protein from each sample was loaded into each well for SDS-PAGE, and the proteins were transferred to nitrocellulose membranes (Protran). Membranes were rocked for 1 h at room temperature in blocking buffer (3% nonfat milk dissolved in TBS-T: 50 mM Tris pH 8, 137.5 mM NaCl, 2.7 mM KCl, 0.08% Tween 20). Membranes were incubated with primary antibody diluted in either 3% milk/TBS-T (tubulin antibody) or 5% BSA/TBS-T (all other antibodies) for 1 h at room temperature. Then membranes were incubated with goat secondary antibody conjugated to HRP (Jackson ImmunoResearch Labs) diluted in 3% milk/TBS-T for 1 h at room temperature. Finally blots were washed in TBS-T, exposed to chemiluminescent substrate (Immobilon) and visualized on an imaging system (AlphaInnotech Fluorchem). Spot densitometry quantitation was performed, by subtracting background signal from each band of interest and dividing that value by the background-subtracted tubulin signal for the same lane, in order to normalize for protein loading.

### RNA interference

4000 cells were plated in each well in a 96 well cell culture plate. Cells were transfected with siRNA (Dharmacon) using RNAiMAX (Invitrogen) in antibiotic-free medium.

### MTS assay

4000 cells were plated in each well in 96 well cell culture plates. The following day, cells were treated with drugs as described in the Results. Every type of treatment was performed in quadruplicate wells. After 48 or 72 h, cell viability was measured using the Promega CellTiter 96 Aqueous Non-Radioactive Cell Proliferation Assay (MTS). 1 to 3 h after the MTS solution was added, the absorbance of the wells was measured in a plate reader at 450 nm.

### Mice

All mice were maintained in a specific pathogen free animal facility at Harvard Medical School. Research was performed in accordance with guidelines and policies as set by the Harvard Medical Area Standing Committee on Animals (approval reference #02460). Mice homozygous for floxed *Pten *exon 5 (*Pten*^*loxp/loxp*^) [31] were crossed to mice transgenic for *Fabpl-Cre *[32]. F1 *Pten^loxp/+^;Fabpl-Cre+ *mice were crossed with *Pten^loxp/loxp ^*mice to obtain *Pten^loxp/loxp^;Fabpl-Cre+ *mice.

### Immunohistochemistry

Murine bladders were fixed in 4% paraformaldehyde/PBS and stored in 70% ethanol until they were embedded in paraffin and sectioned (Harvard Rodent Histopathology Core). Sections were deparaffinized and subject to 0.5% hydrogen peroxide/PBS treatment for 10 min. Antigen retrieval was accomplished by boiling sections in 0.01 M Citrate pH 6 buffer for 10 min in a microwave. Sections were blocked in 3% donkey serum/PBS/0.2% triton-X. Primary antibody was added in blocking solution for 2 h at room temperature or overnight at 4C, followed by washes and incubation with biotinylated secondary antibody for 1 h at room temperature. The Vectastain ABC system (Vector Labs) was used in conjunction with either Vector Red substrate for alkaline phosphatase or 3,3'-diaminobenzidine (DAB) substrate for peroxidase. All sections were further stained with hematoxylin, dehydrated, and mounted with Permount™.

### Transfections

Cells were counted, and 2 × 10^5 ^cells were plated in 60 mm dishes. The following day 2 μg of DNA was mixed with 6 μl of Fugene in Opti-MEM solution (Invitrogen). After 15 min, the mixture was dropped onto cells and left to incubate for 48 h. Cells were then washed with PBS and depending on treatment, some cells were incubated in serum-free DMEM for 24 additional hours. Cells were lysed and subject to western blotting as described above.

### Statistical analyses

All statistical tests (Oneway ANOVA, Tukey-Kramer HSD, and Student's t-test) were done using JMP9 software.

## Results

**Figure 1:**
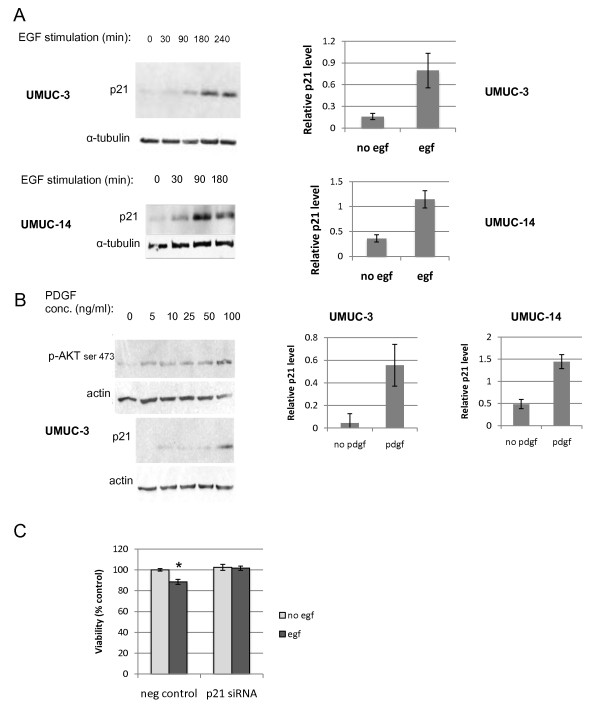
**Growth factors induce p21 and inhibit proliferation in bladder carcinoma cells**. UMUC-3 and UMUC-14 human urothelial carcinoma cell lines were serum starved for 24 hours and then stimulated with growth factors. (A) Cells were lysed at intervals after addition of 10 ng/ml EGF, and proteins were harvested for immunoblotting against p21 and α-tubulin. Quantitation of relative p21 signal normalized to tubulin after 180 minute EGF treatment is shown in the graphs. There were 4 replicates of each treatment for the UMUC-3 cells and 3 replicates of the UMUC-14 cells. Error bars represent S.E.M. (B) UMUC-3 cells were treated with varying concentrations of PDGF for 90 minutes and then were harvested. Lysates were immunoblotted for expression of p21 and phosphorylation levels of AKT at serine 473. Relative levels of p21 normalized to tubulin after 90 minutes of 100 ng/ml PDGF treatment is shown in the graphs for both UMUC-3 and UMUC-14 cells. There were 4 replicates of each treatment. (C) UMUC-3 cells were transfected with control or p21 siRNA and 48 hours later, cells were serum starved for 24 hours. Then 10 ng/ml EGF was added to half of the wells and 24 hours later an MTS assay was run. * significantly different from all other treatments, p = .002.

In our previous studies, we found that deletion of *Pten *in murine bladder epithelium leads to an increase in p21 expression, and that the p21 mediates a significant decrease in cell proliferation [19]. We therefore investigated whether p21 was similarly induced in human urothelial carcinoma cells by signaling through the PI3-kinase pathway. We selected two cell lines: UMUC-3 (PTEN negative) and UMUC-14 (PTEN positive)for our studies. These cell lines were originally derived from two independent urothelial carcinomas. The cells were serum starved for 24 hours in order to attenuate growth factor signaling. Then, we treated cells with two different growth factors that are known to activate PI3-kinase: epidermal growth factor (EGF) and platelet-derived growth factor (PDGF) [33,34]. When treated with EGF, p21 levels increased in both cell lines (Figure 1A). While UMUC-14 cells do express PTEN protein, the cell line is still clearly responsive to growth factor signaling. We also treated both cell lines with PDGF because if the p21 induction was dependent on PI3-kinase, then signaling through multiple growth factor receptors should induce p21. PDGF did induce p21 in both cell types, especially at the concentration of 100 ng/ml (Figure 1B).

It was important to see if the increased p21 in the cells affects cell proliferation, so we transfected the UMUC-3 cells with either control siRNA or p21 siRNA to knock down p21 expression. Two days after transfection, cells were washed in PBS and serum starved for 24 hours. Then EGF (10 ng/ml) was added to some of the wells for 24 hours, and an MTS assay was run to measure cell proliferation. As seen in Figure 1C, EGF treatment actually significantly decreased the number of viable cells, and knock down of p21 prevented this effect of EGF (ANOVA; p = .002), showing that the induction of p21 in response to EGF results in reduced cell proliferation.

**Figure 2:**
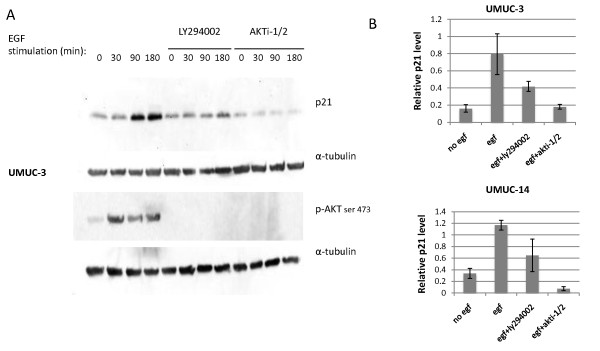
**EGF induces p21 in a PI3-kinase and AKT dependent manner**. (A) UMUC-3 cells were pre-treated for one hour with PI3-kinase inhibitor LY294002 (30 μM) or AKT inhibitor AKTi-1/2 (10 μM) and then stimulated with EGF over a 180 minute time course. Cells were harvested at times shown and probed for expression of p21 and phosphorylated AKT serine 473. (B) Quantitation of relative p21 signal normalized to tubulin after 180 minute EGF treatment in the presence or absence of pretreatment with LY294002 or Akti-1/2 is shown. Four replicates of each treatment were tested. Error bars represent S.E.M.

In order to verify whether this p21 induction was dependent on PI3-kinase and AKT signaling, cells were stimulated with EGF in the presence or absence of LY294002, a PI3-kinase inhibitor, or Akti-1/2, an AKT inhibitor (Figure 2). Western blotting showed that p21 induction did not occur in the presence of AKT inhibitor, and there was very weak p21 induction in the presence of LY294002, indicating that p21 induction was in fact dependent on PI3-kinase and AKT activity. We confirmed that the inhibitors were effective at blocking signaling by testing for AKT phosphorylation at the critical activation site, serine 473 (Figure 2A).

**Figure 3:**
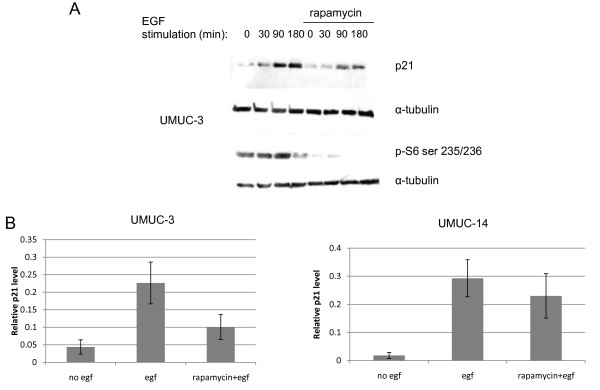
**Induction of p21 is partially dependent on mTOR**. (A) UMUC-3 cells were serum starved for 24 hours and then stimulated with 10 ng/ml EGF in the presence or absence of 1 hour pretreatment with 12.5 nM rapamycin. S6 phosphorylation at serines 235/236 was also measured to verify rapamycin-mediated inhibition of mTOR. (B) Quantitation of relative p21 induction by EGF in the presence of absence of rapamycin in UMUC- and UMUC-14 cells. Error bars represent S.E.M.

AKT is known to cause downstream mTOR activation [1], and since mTOR increases translation of many proteins, we tested if mTOR was responsible for the increased p21 levels in the urothelial cell lines stimulated with growth factors. We pre-treated some of the UMUC-3 cells for one hour with 12.5 nM rapamycin, an mTOR inhibitor, and then stimulated the cells with EGF. Immunoblotting for phosphorylated ribosomal S6 protein, a critical downstream substrate that mediates mTOR's effects on protein synthesis, verified that the rapamycin suppressed mTOR activity (Figure 3A). We found that p21 was still induced by EGF in the presence of rapamycin in the UMUC-14 cells and possibly also in the UMUC-3 cells (Figure 3A and 3B), although to a reduced extent. This indicated that the p21 induction caused by the PI3-kinase pathway is partially mediated by mTOR.

**Figure 4:**
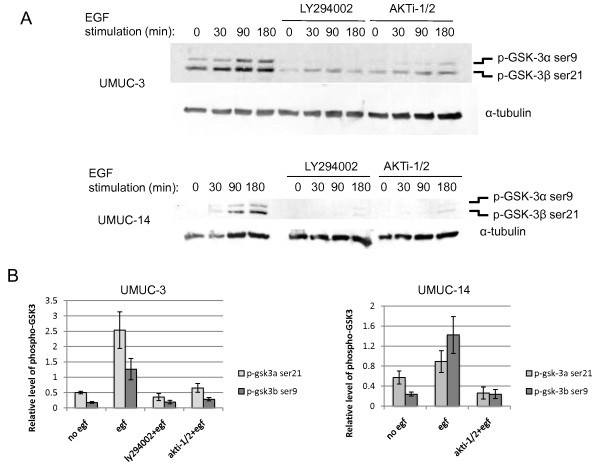
**EGF stimulates inhibitory phosphorylation of GSK-3α and β in a PI3-kinase and AKT dependent fashion**. Phosphorylation of GSK-3α and β at serines 9 and 21 respectively was tested by immunoblotting of (A) UMUC-3 and UMUC-14 cells that had been serum starved for 24 hours and then stimulated with 10 ng/ml EGF. Some cells were also pre-treated with 30 μM LY294002 or 10 μM AKTi-1/2 in order to verify that phosphorylation was dependent on PI3-kinase and AKT activity. (B) Quantitation of relative phospho-GSK-3α and β levels normalized to tubulin is shown for UMUC-3 and UMUC-14 cells. Four replicates were tested. Error bars represent S.E.M.

Since the PI3-kinase/AKT signaling pathway is known to inhibit GSK-3β activity [16], and GSK-3β has been reported to cause the degradation of other proteins such as β catenin [17] and SMAD1 [35], we decided to investigate its involvement in p21 regulation in bladder cells. First, we examined if PI3-kinase/AKT signaling results in GSK-3 inhibition in UMUC-3 and UMUC-14 cells. While most studies have examined the role of GSK-3β in this pathway, the function of the GSK-3α isoform is less clear, so we investigated the status of both isoforms. AKT has been shown to directly phosphorylate GSK-3α and β at serines 9 and 21 [16], respectively, resulting in an inhibition in GSK-3 activity. Treatment of both UMUC-3 and UMUC-14 cells with EGF resulted in increased phosphorylation of GSK-3α and β at serine 9 and 21(Figure 4A and 4B). In both cell types, the phosphorylation of GSK-3α and β was PI3-kinase and AKT dependent. This result confirmed that GSK-3 is inhibited by PI3-kinase/AKT in human bladder cells and led to our subsequent investigation to determine if the GSK-3 inhibition affects p21 levels.

**Figure 5:**
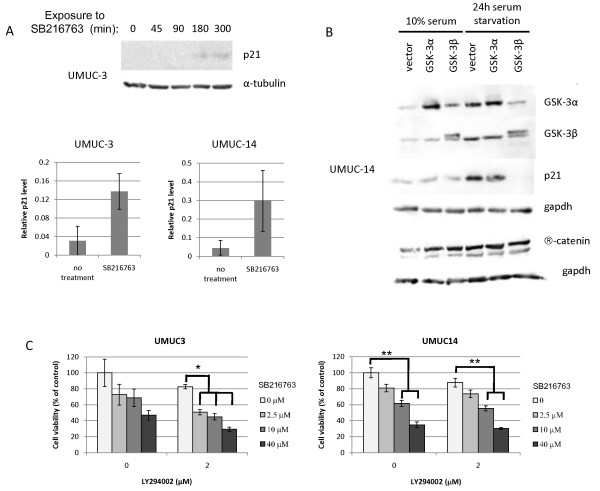
**GSK-3 regulates p21 expression, and inhibition of GSK-3 reduces cell viabiliity in bladder carcinoma cell lines**. (A) UMUC-3 cells were serum-starved, then treated with GSK-3 inhibitor SB216763 (2.5 μM) over a time course and lysates were immunoblotted for p21 expression. Below, p21 levels normalized to tubulin are shown for UMUC-3 and UMUC-14 cells after treatment for five hours with 2.5 μM SB216763 compared to cells without drug treatment. (B) UMUC-14 cells were transfected with GSK-3α and β expression vectors, and then 48 hours later were serum starved for an additional 24 hours. Cells were subsequently lysed and immunoblotted for GSK-3α and β, p21, β-catenin, and gapdh. (C) An MTS cell viability assay was run on UMUC-3 and UMUC-14 cells treated with SB216763, LY294002, or a combination of the two. UMUC-3 cells were tested by MTS assay after 72 hour drug treatment, and UMUC-14 cells were tested after 48 hour treatment. Asterisks refer to results of Tukey-Kramer post-hoc test comparing cell viabilities at the same concentration of LY294002. *p < .0001; **p < .001.

In order to ascertain if GSK-3 inhibition causes an increase in p21 in bladder cells, UMUC-3 and UMUC-14 cells were serum starved for 24 hours to suppress PI3-kinase signaling and thereby promote GSK-3 activity. The cells were then treated with the GSK-3 inhibitor SB216763 over five hours. Levels of p21 were initially low after serum starvation, but increased in response to SB216763 treatment (Figure 5A), indicating that GSK-3 activity negatively regulates p21 expression. UMUC-3 cells contain a mutated p53, so the p21 induction in response to SB216763 is not p53-dependent in these cells.

In order to more directly test the effects of GSK-3α and β individually on p21, we transiently transfected GSK-3 expression vectors into UMUC-14 cells for 48 hours. The exogenous GSK-3β has a slightly higher molecular weight than does endogenous GSK-3β due to the presence of a C-terminal HA tag (Figure 5B). When p21 levels were tested in the presence of 10% fetal calf serum, no effects of GSK-3 expression on p21 were evident. However, when transfected cells were serum starved for 24 hours in order to minimize GSK-3 inhibition by AKT, exogenous expression of GSK-3β resulted in a massive decrease in p21 levels (Figure 5B). GSK-3α had a lesser effect but also appeared to diminish p21 levels in experimental replicates. This indicates that GSK-3β downregulates p21, but that activity is inhibited in the presence of serum and an active PI3-kinase pathway. Interestingly, β catenin levels were unaffected by exogenous GSK-3 expression (Figure 5B), which suggests that β catenin did not play a role in the induction of p21.

The ability of the GSK-3 inhibitor SB216763 to induce p21 led us to speculate that SB216763 should inhibit urothelial carcinoma cell proliferation. We further hypothesized that the combination of SB216763 with a PI3-kinase inhibitor such as LY294002 should more effectively inhibit cell proliferation and/or induce cytotoxicity than LY294002 alone. In order to test these hypotheses, we treated UMUC-3 and UMUC-14 cells with various concentrations of SB216763 in the presence or absence of LY294002. After 48 hours (for UMUC-14 cells) or 72 hours (for UMUC-3 cells), we performed an MTS assay to measure cell viability (Figure 5C). The UMUC-3 cells had a longer drug treatment because they showed relatively little cytotoxicity at 48 hours. After 72 hours, the UMUC-3 cells did not show significant decreases in viability due to treatment with LY294002 alone or SB216763 alone, even at the highest concentrations used in this assay, although there was a trend toward decreased viability for both drugs. A combination of the two drugs was most effective for cell cytotoxicity; 2 μM LY294002 plus SB216763 at any tested concentration caused significant decreases in UMUC-3 cell viability compared to 2 μM LY294002 alone (Oneway ANOVA; p < .0001). While treatment of the UMUC-14 cells with 2 μM LY294002 alone had no significant effect on cell viability compared to untreated cells, there was a dose-dependent cytotoxicity response to SB216763 alone (Oneway ANOVA; p < .0001), and there was a significant decrease in cell viability at 10 μM SB216763 (Tukey-Kramer HSD; p = .0004) and at 40 μM SB216763 (Tukey-Kramer HSD; p < .0001) compared to the untreated control cells. A combination of the two drugs was once again more effective for cytotoxicity; when 2 μM LY294002 was added to the UMUC-14 cells together with 10 μM SB216763, there was a significant further decrease in cell viability compared to LY294002 alone (Oneway ANOVA; p < .0001; Tukey-Kramer HSD; p = .0007). In addition, there was significantly more cytotoxicity at 40 μM SB216763 when comparing cells in the presence of LY294002 compared to the cells that received no LY294004 (Student's t-test; p = .02). This suggests that increased inhibition of cell viability can be attained with a combination of the LY294002 PI3-kinase inhibitor and the SB216763 GSK-3 inhibitor.

**Figure 6:**
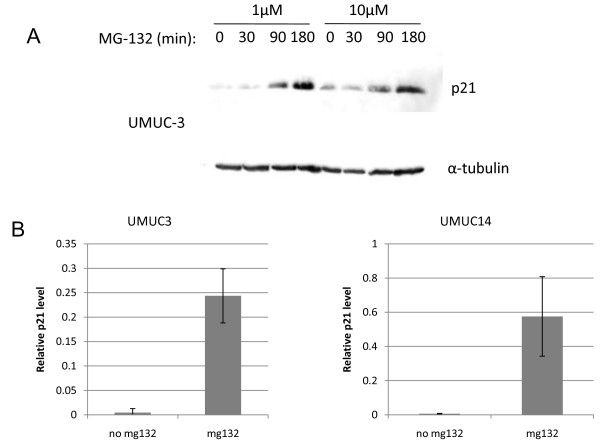
**Proteasome inhibition causes an increase in p21**. (A) UMUC-3 cells were serum starved for 24 hours and then treated with 1 μM and 10 μM MG-132 over a 180 minute time course. Cells were lysed and immunoblotted for p21 and tubulin. (B) Quantitation of relative p21 levels normalized to tubulin after 180 minutes treatment with 1 μM MG-132 is shown for UMUC-3 and UMUC-14 cells. Four replicates were tested. Error bars represent S.E.M.

GSK-3β is known to directly phosphorylate substrates leading to ubiquitylation and subsequent proteasome dependent degradation of those proteins. It is unclear, however, if p21 is targeted for proteasomal destruction in these cells. If GSK-3 was regulating p21 in this manner in these cell lines, then we should expect to see evidence that the proteasome regulates p21 levels. We found that serum starvation to activate GSK-3, followed by proteasome inhibition using MG-132 leads to an increase in p21 in both UMUC-3 and UMUC-14 cells (Figure 6), suggesting that p21 levels are regulated by proteasomal degradation.

**Figure 7:**
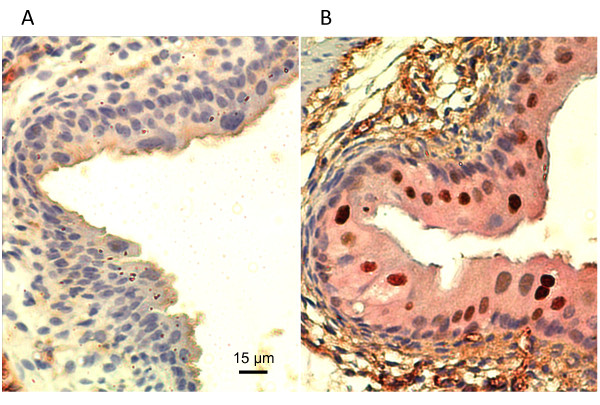
***Pten *deletion causes an increase in cytoplasmic phosphorylated GSK-3 α/β ser9/21 in murine bladder epithelium**. Sections of bladder from (A) three day old wild-type *Pten*^*loxp/loxp *^mice and (B) *Pten *deficient *Fabpl-Cre; Pten*^*loxp/loxp *^mice were double immunostained for p21 (brown) and p-GSK-3α/β ser9/21 (pink). Photos were originally taken at 200 × magnification.

Given the evidence that GSK-3β inhibition causes elevated p21 levels in PI3-kinase stimulated human urothelial cells, we examined if GSK-3β is also inhibited in mice that are conditionally deficient for *Pten *(Fabpl-Cre;*Pten*^loxp/loxp^)in bladder urothelium. These mice were previously shown to have elevated levels of nuclear p21 in urothelium [19]. Immunohistochemical staining of *Pten *deficient mouse bladders and their wild-type littermates showed not only higher levels of p21 positive cells in the bladder, but also greatly increased cytoplasmic staining of phospho-GSK3α and β at serines 9 and 21 (Figure 7). This finding that GSK-3 exhibits large levels of inhibitory phosphorylation at sites that are known targets for AKT is consistent with the idea that GSK-3 inhibition contributes to elevated p21 levels in the *Pten*-deficient mouse bladder.

## Discussion

Deletion of *Pten *causes increased tumorigenesis in a mouse model of bladder cancer [19], and there is a great deal of evidence of decreased PTEN expression in human bladder carcinomas [4-7]. However, those same studies on human tumors have suggested that testing PTEN expression levels in urothelial tumors is not useful for clinical management, since it does not predict disease progression or survival. While one reason for this discrepancy may be that multiple mechanisms besides diminished PTEN lead to overstimulation of the PI3-kinase/AKT pathway [36], we propose that an additional consideration may be the increased expression of p21 in bladder epithelium when the PI3-kinase pathway is activated. We have now shown in both the mouse *in vivo *and in human urothelial cell lines that stimulation of the PI3-kinase/AKT pathway leads to an increase in p21 levels. Our studies in the mouse [19] and multiple studies by others in human and rat bladder cancer cell lines [37-39] have shown that induction of p21 inhibits cell proliferation. Therefore, when bladder cells acquire mutations that cause overactive PI3-kinase/AKT signaling, the ensuing p21 expression provides a protective response by suppressing proliferation, and it may delay tumorigenesis. This p21 induction from PI3-kinase signaling could partially explain why early stage and low grade bladder tumors demonstrate elevated levels of p21 [25,26], whereas advanced tumors have reduced p21 expression [25,28,29]; the p21 provides a protective function and suppresses tumor growth. Future studies should examine *both *the PTEN/PI3-kinase pathway and p21 expression levels in human tumors simultaneously in order to more accurately predict the course of disease.

In this paper, we examined both a PTEN negative cell line, UMUC-3, and a PTEN positive cell line, UMUC-14, and found similar results in terms of mechanism of p21 induction in both cell types. This does not contradict our initial finding in mice that p21 induction occurs in the *Pten *deficient bladders, because in this paper, the growth factors such as EGF and PDGF may have overwhelmed PTEN's ability to counteract the PI3-kinase stimulation in the UMUC-14 cells. It is possible that the p21 levels may eventually fall again in the PTEN positive UMUC-14 cells as PTEN downregulates the PI3-kinase signaling pathway. It was our observation that in the *Pten *deficient animals, the p21 levels remained elevated for over a year. It is worth noting here that the UMUC-3 cells usually exhibited superior p21 induction and GSK-3 phosphorylation compared to the UMUC-14 cells, which is consistent with the PTEN negative status of UMUC-3 cells. Nevertheless, these pathways were functional in the UMUC-14 cells. Our finding that the UMUC-14 cells were initially more susceptible to LY294002 cytotoxicity than the UMUC-3 cells was surprising, since UMUC-14 cells show low levels of AKT activation (8). A recent study indicated that UMUC-14 cells were also more susceptible than UMUC-3 cells to growth inhibition by mTOR inhibitor RAD001 [40], hinting at a possible "addiction" to downstream signaling in the PI3-kinase/AKT pathway. In the future, these studies should be performed in primary human urothelial cells instead of cell lines, to rule out the possibility that the mechanisms observed here do not accurately reflect what occurs in normal urothelial cells. In addition, cell culture does not take into effect the possible role of differentiation in affecting p21 levels.

**Figure 8:**
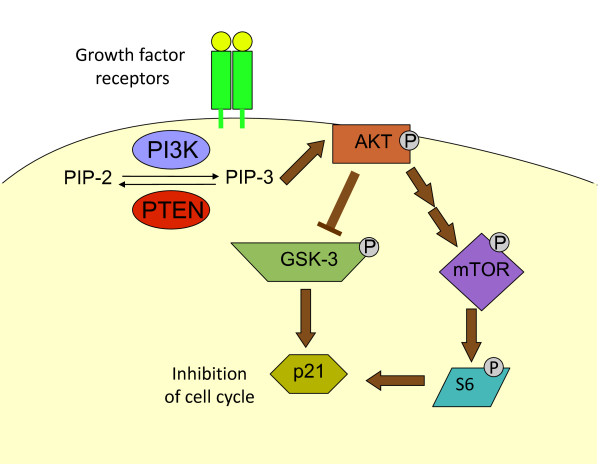
**A model for p21 regulation by the PI3-kinase/PTEN signaling pathway**. Growth factor signaling stimulates PI3-kinase and induces an increase in PIP-3 levels at the plasma membrane; PTEN converts PIP-3 back to PIP-2. An increase in PIP-3 causes AKT activation. AKT phosphorylates GSK-3 causing the inhibition of GSK-3. In the absence of GSK3 activity, p21 levels rise. AKT also activates mTOR, causing an increase in S6 phosphorylation and translation of proteins, which in turn leads to an increase of p21. The p21 causes an inhibition of cell cycle progression.

Our study has shown that GSK-3β may be an important mechanism by which p21 levels are regulated in the context of PI3-kinase/AKT signaling (Figure 8). It is unclear, however, if GSK-3β is directly regulating p21 or if the mechanism is indirect. GSK-3β is known to directly phosphorylate substrates such as β-catenin, cyclin D1, and cyclin D2, leading to ubiquitylation and subsequent proteasome-dependent degradation of those proteins [41-43]. It is certainly possible that GSK-3β regulates p21 in this fashion [44] in the bladder cells. We found that proteasome inhibition of the UMUC-3 cells leads to an increase in p21, suggesting that p21 levels are regulated by proteasomal degradation. Ubiquitin-independent proteasomal degradation of p21 has also been described [45]. One study by Li et al. (2002) [46] suggested that AKT could directly phosphorylate p21 at Thr 145 to stabilize the protein, but we were unable to find evidence that p21 was phosphorylated at this residue. We also found evidence that PI3-kinase signaling partially increases p21 levels through mTOR activity, most likely due to enhanced protein synthesis. While work remains to be done in this area, our studies have defined two pathways, GSK-3β and mTOR, by which p21 levels are regulated in urothelial cells during PI3-kinase/AKT signaling.

A fuller understanding of the mechanisms by which p21 is upregulated by PI3-kinase/AKT signaling will allow potential therapeutic intervention to increase p21 expression in tumors that have lost it. Although GSK-3β is generally considered to negatively regulate cell growth, GSK-3β inhibitors have been found to reduce colon and ovarian tumor cell growth [47]; these drugs may be appropriate in bladder cancer because of their effects on p21. It will be helpful to examine GSK-3β activation levels in urothelial tumors in order to assess the possible utility of GSK-3β inhibitors. This pathway should also be considered where urothelial cancer patients are treated with PI3-kinase inhibitors, since the decrease in p21 as a result of the drugs may limit the effectiveness of the therapy. Our studies suggest that combination therapy with PI3-kinase and GSK-3 inhibitors could be more effective than PI3-kinase inhibitors alone.

## Conclusions

This is the first description of GSK-3β mediated regulation of p21 in bladder cells. We have found evidence that inhibition of GSK-3β either through activation of the PI3-kinase/AKT signaling pathway or through the use of pharmacological inhibitors of GSK-3β leads to an increase in p21 levels, while an overexpression of GSK-3β in cells with reduced PI3-kinase/AKT signaling leads to a decrease in p21. Signaling through mTOR also contributes to the induction of p21. This information will be useful in predicting outcomes and tailoring chemotherapy for bladder cancer patients.

## Competing interests

The authors declare that they have no competing interests.

## Authors' contributions

LY conceived of the study, carried out most of the cell culture work, MTS assays, immunohistochemistry, and immunoblotting, and wrote the manuscript. NY, CB, and AD carried out some of the cell culture work and immunoblotting, and quantified immunoblot signals. All authors reviewed and approved the final version of the manuscript.

## Pre-publication history

The pre-publication history for this paper can be accessed here:

http://www.biomedcentral.com/1471-2490/11/19/prepub
